# Evidence for the involvement of gamma delta T cells in the immune response in Rasmussen encephalitis

**DOI:** 10.1186/s12974-015-0352-2

**Published:** 2015-07-19

**Authors:** Geoffrey C. Owens, Kate L. Erickson, Colin C. Malone, Calvin Pan, My N. Huynh, Julia W. Chang, Thabiso Chirwa, Harry V. Vinters, Gary W. Mathern, Carol A. Kruse

**Affiliations:** Department of Neurosurgery, David Geffen School of Medicine at University of California, Los Angeles, 300 Stein Plaza, Ste. 562, Los Angeles, CA 90095-6901 USA; Department of Human Genetics, David Geffen School of Medicine at University of California, Los Angeles, Los Angeles, USA; Department of Pathology and Laboratory Medicine, David Geffen School of Medicine, University of California, Los Angeles, Los Angeles, USA; Department of Neurology, David Geffen School of Medicine, University of California, Los Angeles, Los Angeles, USA; Intellectual and Developmental Disabilities Research Center, David Geffen School of Medicine, University of California, Los Angeles, Los Angeles, USA; Brain Research Institute, David Geffen School of Medicine, University of California, Los Angeles, Los Angeles, USA; Mattel Children’s Hospital, David Geffen School of Medicine, University of California, Los Angeles, Los Angeles, USA

**Keywords:** Rasmussen encephalitis, Brain, Inflammation, Focal cortical dysplasia, T cells, Gamma delta T cells, T cell receptor, CDR3

## Abstract

**Background:**

Rasmussen encephalitis (RE) is a rare neuroinflammatory disease characterized by intractable seizures and progressive atrophy on one side of the cerebrum. Perivascular cuffing and clusters of T cells in the affected cortical hemisphere are indicative of an active cellular immune response.

**Methods:**

Peripheral blood mononuclear cells (PBMCs) and brain-infiltrating lymphocytes (BILs) were isolated from 20 RE surgery specimens by standard methods, and CD3^+^ T cell populations were analyzed by flow cytometry. Gamma delta T cell receptor spectratyping was carried out by nested PCR of reversed transcribed RNA extracted from RE brain tissue, followed by high resolution capillary electrophoresis. A MiSeq DNA sequencing platform was used to sequence the third complementarity determining region (CDR3) of δ1 chains.

**Results:**

CD3^+^ BILs from all of the RE brain specimens comprised both αβ and γδ T cells. The median αβ:γδ ratio was 1.9 (range 0.58–5.2) compared with a median ratio of 7.7 (range 2.7–40.8) in peripheral blood from the same patients. The αβ T cells isolated from brain tissue were predominantly CD8^+^, and the majority of γδ T cells were CD4^−^ CD8^−^. Staining for the early activation marker CD69 showed that a fraction of the αβ and γδ T cells in the BILs were activated (median 42 %; range 13–91 %, and median 47 %; range 14–99 %, respectively). Spectratyping T cell receptor (TCR) Vδ1-3 chains from 14 of the RE brain tissue specimens indicated that the γδ T cell repertoire was relatively restricted. Sequencing δ1 chain PCR fragments revealed that the same prevalent CDR3 sequences were found in all of the brain specimens. These CDR3 sequences were also detected in brain tissue from 15 focal cortical dysplasia (FCD) cases.

**Conclusion:**

Neuroinflammation in RE involves both activated αβ and γδ T cells. The presence of γδ T cells with identical TCR δ1 chain CDR3 sequences in all of the brain specimens examined suggests that a non-major histocompatibility complex (MHC)-restricted immune response to the same antigen(s) is involved in the etiology of RE. The presence of the same δ1 clones in CD brain implies the involvement of a common inflammatory pathway in both diseases.

**Electronic supplementary material:**

The online version of this article (doi:10.1186/s12974-015-0352-2) contains supplementary material, which is available to authorized users.

## Background

Rasmussen encephalitis (RE) is a rare pediatric neurological disease with an estimated incidence in children under the age 18 years of 2–3 per 10 million [1–3]. The acute phase of the disease is characterized by intense uncontrolled partial or generalized seizures, and MRI FLAIR imaging often shows inflammation in one cerebral hemisphere [3]. As the disease progresses, unilateral loss of cerebral tissue leaves the patient with severe hemiparesis and other neurological deficits. Corticosteroids may provide short term benefit but ultimately fail to halt the disease. Early treatment with tacrolimus or intravenous immunoglobulins may stabilize the neurological deterioration, but they do not reverse the intractable epilepsy [2]. An inflammatory response involving T cells and activated microglia confined to the affected hemisphere appears to be the cause of the clinical symptoms. However, what precipitates the immune response is not known. Several types of Herpesviridae have been detected in surgical brain specimens from RE patients; however, to date, there is no consistent evidence for a pathogen that is common to all RE cases [4–7]. Likewise, autoantibodies have been described in RE cases indicative of an autoimmune disease, but autoantibodies have not been found in all RE cases [8–11].

The observation of polarized granzyme B-containing CD8^+^ T cells in brain parenchyma in close proximity to neurons and astrocytes has pointed to a role for major histocompatibility complex (MHC) class I-restricted CD8^+^ cytotoxic T cells in RE [12]. The cytotoxic T cells are likely reacting to foreign or self-antigens displayed by neurons and astrocytes in the affected cerebral hemisphere. Confinement of the T cells to one cerebral hemisphere suggests that the initial inflammatory reaction may have been spatially restricted. Such a reaction would have triggered a localized innate immune response by brain resident macrophages (microglia) and could have led to the recruitment of nonresident non-MHC-restricted immune cells, such as natural killer cells and γδ T cells followed by primed MHC-restricted αβ T cells. In the present study, we document for the first time the presence of clonally restricted γδ T cells in brain tissue from RE patients, indicating a role for this T cell subtype in the inflammatory response in RE.

## Methods

### RE patient cohort and clinical variables

Under the University of California, Los Angeles, Institutional Review Board (UCLA IRB) approval (IRB #11-00030), brain tissue and blood were collected at surgery as part of UCLA’s Pediatric Epilepsy Surgery Program. For cases that were not treated at UCLA, tissue and blood were provided to UCLA under the auspices of the UCLA IRB approved Rare Brain Disease Tissue Bank (IRB# 13-001213). All of the patients or their parents or legal guardians provided informed consent for the use of the surgical remnant and blood for research purposes. All specimens were collected using the same standard operating procedures (SOPs); SOPs were provided by UCLA to the contributing institutions. De-identified patient information was collected with informed consent including age at seizure onset, age at surgery, gender, and affected cerebral hemisphere.

### Isolation of peripheral blood lymphocytes and brain-infiltrating lymphocytes

Peripheral blood mononuclear cells (PBMCs) were isolated by density gradient centrifugation using Ficoll-Paque PLUS (GE Healthcare, Piscataway, NJ). Brain-infiltrating lymphocytes (BILs) were isolated from collagenase-treated brain tissue by fractionation on a step gradient. Briefly, brain tissue was diced manually on ice in dissociation solution (HBSS with 20 mM HEPES pH 7.0, 5 mM glucose, and 50 U/ml penicillin/streptomycin). Tissue fragments were incubated with agitation in dissociation solution containing 0.5 mg/ml type IV collagenase (Worthington Biochemical Corp., Lakewood, NJ) and 5 % filtered human serum (Mediatech Inc., Manassas, VA) at 37 °C for 3 h or at room temperature overnight. The dissociated tissue was fractionated on a 30:70 % Percoll® (SigmaAldrich, St. Louis, MO) step gradient in RPMI containing 20 mM HEPES. PBMCs and BILs were cryopreserved in 90 % human serum/10 % DMSO.

### Analysis of lymphocytes by flow cytometry

Phenotypic data were acquired on an analytical LSRII flow cytometer (Becton Dickinson, San Jose, CA). The following antibodies were used: APC-efluor® 780-conjugated CD3 (clone UCHT1; eBioscience Inc., San Diego, CA), PE/Cy7-conjugated CD4 (clone SK3; eBioscience Inc.), PerCP/Cy5.5-conjugated CD8 (clone RPA-T8; eBioscience Inc.), APC-conjugated T cell receptor (TCR) αβ (clone IP26; eBioscience Inc.), FITC-conjugated TCR γδ (clone B1.1; eBioscience Inc.), PE-conjugated CD69 (clone FN50; eBioscience Inc.), PE-conjugated TCR Vδ2 (clone B6; Biolegend), and FITC-conjugated TCR Vδ1 (Clone TS8.2; GeneTex Inc., Irvine, CA). Data were analyzed with FlowJo software (TreeStar Inc., Ashland, OR); plots were exported to CorelDRAW X6 (Corel Corporation, Ottawa, Canada). Statistical analyses and graphing utilized R-project programs (www.r-project.org).

### Spectratyping T cell receptor Vδ chains

Total RNA was purified from flash frozen blocks of involved tissue consisting of mostly cortical gray matter (~50 mg) using Trizol™ (Life Technologies, Carlsbad, CA) followed by column purification (Qiagen, Valencia, CA). RNA was reverse transcribed (Qiagen), and PCR reactions were carried out in an Applied Biosystems (ABI) GeneAmp® PCR system 9700 using AccuPrime™ Taq polymerase (Life Technologies). The same primer sets described by Dechanet et al. [13] were used; forward primers were unique to Vδ1, Vδ2, and Vδ3, respectively, and two nested reverse primers were located in the constant region. The sequences of each primer are as follows: Vδ1 5′ CTGTCAACTTCAAGAAAGCAGCGAAATC 3′; Vδ2 5′ TACCGAGAAAAGGACATCTATGGC 3′; Vδ3 5′GGGGATAACAGCAGATCAGAAGGT 3′; Cδ1.1 5′ TGGGAGAGATGCAATAGCAGGATC 3′; Cδ1.2 5′ ACGGATGGTTTGGTAGAGGCTGA 3′. The Cδ1.2 primer was end-labeled with 6-carboxyfluorescein (FAM). The cycling conditions were as follows: 94 °C 2 min followed by 40 cycles of 94 °C 45 s, 60 °C 45 s, 68 °C 45 s, and a run off step at 68 °C for 4 min. For the second round of PCR, the number of cycles was reduced to 35 cycles. FAM-labeled products were separated on an ABI 3730 Capillary DNA Analyzer (Life Technologies), and peak areas were calculated using ABI Peak Scanner Software 2 (Life Technologies). In order to compare the relative amounts of each Vδ chain-specific fragment between samples, areas of individual peaks in each sample were normalized to the total peak area in each sample. Data were clustered and displayed as a heat map using the GENE-E bioinformatics package (www.broadinstitute.org).

### DNA sequencing T cell receptor Vδ CDR3s

First round Vδ2 and Vδ3 PCR fragments that yielded a single FAM-labeled fragment were re-amplified with the appropriate chain-specific forward primer and an unlabeled Cδ1.2 reverse primer. PCR products were treated with ExoSAP-IT (Affymetrix, Inc. Santa Clara, CA) and sequenced by the Sanger sequencing method using BigDye® cycle sequencing chemistry (Life Technologies). Sequences were analyzed using International Immunogenetics Information System (IMGT)/V-QUEST, the IMGT web portal for analysis of T cell receptor and immunoglobulin sequences [14]. To sequence Vδ1 fragments, adaptors were added to the first round PCR products in the second PCR step, and the resulting DNA fragments were separated by agarose gel electrophoresis and purified (Qiagen). The following primers were used (TCR sequences are underlined): Vδ1 adaptor (forward primer) 5′ GAGACAGTCGTCGGCAGCGTCAGATGTATAACTGTCAACTTCAAGAAAGCAGGAAATC 3′; Cδ1.2 adaptor (reverse primer) 5′ GTCTCGTGGGCTCGG AGATGTGTATAAGAGACAGACGGATGGTTTGGTAGAGGCTGA 3′. Nextera XT indices (Illumina, San Diego, CA) were added by tagging PCR using a KAPA Hifi PCR kit (KAPA Biosystems, Boston, MA), and the PCR products were purified using the Agencourt AMPure XP system (Beckman Coulter, Brea, CA). Fragments were sized on a Bioanalyzer instrument (Agilent Technologies, Inc. Santa Clara, CA), quantified by qPCR (KAPA library quant kit), and pooled. Libraries were sequenced on a MiSeq desktop sequencer using V2 2 × 250 bp chemistry (Illumina). IMGT/HighV-QUEST, the IMGT web portal for high throughput analysis of T cell receptor and immunoglobulin sequences was used to curate all of the sequence data [14]. Scripts were written in R and Python to collate and enumerate the sequences. The δ1-specific sequences from cortical dysplasia (CD) brain samples were generated as described above. In the second PCR step, CDR3 clone-specific forward primers were used together with an extended Cδ1.1 primer, 5′ CTGGGAGAGATGACAATAGCAGGATCAAACTCTG 3′. The following forward primers were used: CDR3 ALGDSIPRRIAYTDKLI, 5′ GCTCTTGGGGATTCCATTCCTAGGAGGATAGCGTACACCGATAAACTCATC 3′; CDR3 ALGGLGTGGYAYTDKLI, 5′ GCTCTTGGGGGGCTAGGTACTGGGGGATACGCCTACACCGATAAACTCATC 3′; CDR3 ALGVPPRPSLYWGIGSLGSYTDKLI, 5′ GCTCTTGGGGTCCCGCCTCGACCTTCCCTCTACTGGGGGATAGGAAGCTTGGGCTCGTACACCGATAAACTCATC 3′. The cycling conditions were as follows: 94 °C 2 min followed by 35 cycles of 94 °C 45 s, 68 °C 90 s, and a run off step at 68 °C for 4 min. PCR products were gel purified and sequenced by the Sanger sequencing method using BigDye® cycle sequencing chemistry (Life Technologies). Sequences were aligned in Seaview [15].

### Immunocytochemistry

Surgical tissue blocks were fixed in freshly prepared 4 % paraformaldehyde in phosphate-buffered saline (PBS) for 24–48 h, then cryoprotected by immersion overnight at 4 °C in 20 % sucrose-PBS followed by 30 % sucrose-PBS. Blocks were then frozen with powdered dry ice and stored at −80 °C. Immunostaining was performed on free-floating 30-μm cryostat-cut sections by first blocking in PBS with 5 % normal goat serum (Vector Laboratories, Burlingame, CA) and 0.3 % Triton X-100 for 1 h. Sections were then incubated in mouse anti-human TCR pan γδ (clone 5A6.E9, 1:100, Thermo Scientific, Waltham, MA) and an anti-human CD3 rabbit polyclonal antibody (1: 100, Dako North America, Inc. Carpinteria, CA) overnight at 4 °C followed by incubation in Alexa Fluor® 488 goat anti-rabbit and Alexa Fluor® 568 goat anti-mouse secondary antibodies (1:1000, Life Technologies) for 1 h at room temperature. Sections were mounted on slides and cover-slipped using ProLong® Gold anti-fade reagent containing DAPI (Life Technologies). Fluorescent images were acquired with an Olympus spinning disk confocal microscope (Olympus America, Inc., Center Valley, PA) controlled by SlideBook™ image acquisition and analysis software (Intelligent Imaging Innovations, Inc. Denver CO). Images were transferred to CorelDRAWX6 (Corel Corporation). Sections (5 μm) of paraffin-embedded involved tissue were deparaffinized and microwaved for 20 min in buffered citrate (10 mM, pH 6.0) to retrieve antigens. After blocking for 1 h (Impress Kit, Vector Laboratories, Burlingame, CA), sections were incubated overnight at 4 °C with rabbit anti-human CD3 (1:800, Dako) or mouse anti-human CD69 (clone CH11, 1:50, Abcam, Cambridge, MA). Peroxidase-conjugated anti-rabbit or anti-mouse secondary antibodies (1: 300; Impress Kit, Vector Laboratories) were added for 1 h at room temperature, followed by incubation with 3,3′-diaminobenzidine (DAB) substrate (MP Biomedicals, Santa Ana, CA) and subsequent counterstaining with hematoxylin. Omission of primary antibodies served as negative controls. Images of the entire sections were acquired with an Aperio ScanScope XT scanner (Aperio, Vista CA) and transferred to CorelDRAWX6 (Corel Corporation).

## Results

### RE patient cohort and collection of surgical specimens

Table 1 provides details of the RE patient cohort used in this study. There were no exclusion criteria. Blood was drawn from the arterial line prior to initiating the surgery. Brain tissue was placed into ice-cold Hibernate® medium immediately after resection. Samples of brain tissue and blood were sent to UCLA in shipping containers that kept the samples at 4 °C.

**Table 1:** Clinical data from 20 RE patients

Case ID	Gender	Age at seizure onset (year)	Age at surgery (year)	Hemisphere
RECP20	F	3	6.1	L
RECP21	M	8	11.9	L
RECP24	F	1.3	2.1	L
RECP25	M	8.3	14.9	L
RECP26	F	6	14.4	L
RECP27	F	7.8	8	R
RECP28	F	9	10	L
RECP29	M	10	13	R
RECP30	F	6	7.9	R
RECP31	M	6	9	R
RECP32	F	8	11	L
RECP33	M	4.3	5.8	L
RECP34	M	9	10.9	R
RECP35	F	4.5	6	L
RECP36	M	6	8	R
RECP37	F	3	3.3	R
RECP39	M	11	12	R
RECP42	F	3	4	L
RECP43	F	7	9.4	L
RECP46	F	6.2	6.7	L

### Brain-infiltrating CD3^+^ cells are predominantly of CD8^+^ αβ^+^ and CD4^−^ CD8^−^ γδ^+^ T cells

To isolate BILs, fresh surgical specimens were digested with collagenase and fractionated on Percoll™ step gradients. CD3^+^ lymphocytes were compared with those in peripheral blood collected from the patients at the time of surgery. Cells were isolated and immunostained at different times because specimens were acquired over a period of 30 months. However, every effort was made to standardize all of the procedures. The yield of BILs varied between samples and was largely dependent upon the amount of tissue obtained.

**Fig. 1:**
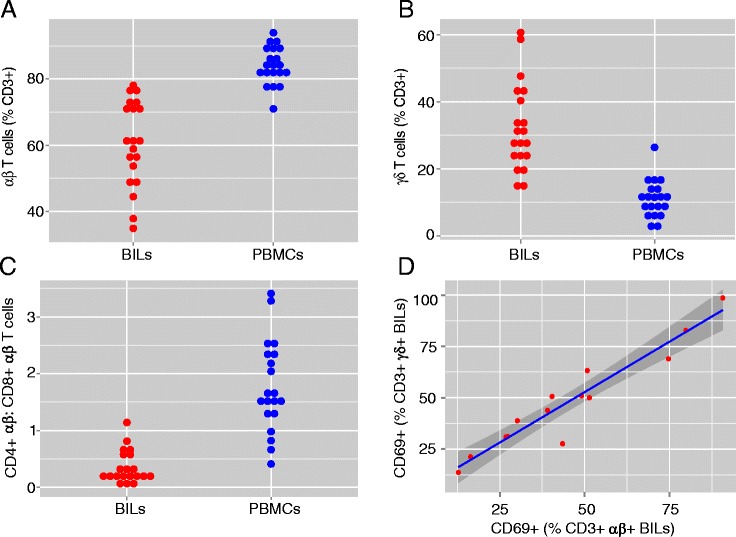
Phenotypic analysis of CD3^+^ T cell subsets in brain-infiltrating lymphocytes (BILs) and in peripheral blood mononuclear cells (PBMCs) from RE patients. The proportion of each T cell subtype as a percentage of the number of CD3^+^ cells gated in each sample is shown. **a** Stacked dot plot showing the percent of αβ T cells in BILs (*red dots n* = 20) compared with PBMCs (*blue dots*) from the same cases (*p* = 0.0001, pair-wise Wilcoxon signed-rank test). **b** Stacked dot plot showing the percent of TCR γδ T cells in BILs compared with PBMCs from the same patients (*p* = 0.0001, pair-wise Wilcoxon signed-rank test). **c** Stacked dot plot showing the ratio of CD4^+^ αβ:CD8^+^ αβ T cells in BILs compared with the corresponding PBMCs (*p* = 0.0001, pair-wise Wilcoxon signed-rank test). **d** Plot of the percent of CD69+ αβ T cells versus CD69+ γδ T cells in BIL fractions (*n* = 14; *r* = 0.9547, CI 0.8595, 0.9859, *p* < 0.0001). Additional file 1: Table S1 provides the quantitative data

BILs and PBMCs were gated on CD3, CD4, CD8, αβ, and γδ T cell markers. In each case, the percent of αβ T cells was higher in the PBMC compared to the BIL fraction (Fig. 1a); however, BILs contained significantly more γδ T cells, which were mainly CD4^−^ CD8^−^ (Fig. 1b; Additional file 1: Table S1). The median ratio of αβ:γδ T cells was 1.9 (range 0.58–5.2) in the BIL fractions, compared with a median ratio of 7.7 (range 2.7–40.8) in PBMCs (Additional file 1: Table S1). αβ T cells were predominantly CD8^+^ in the BIL populations, confirming previous immunocytochemical studies [12, 16–18] (Fig. 1c). The percentage of γδ T cells in the BIL fractions was not significantly correlated with the age at which the patient presented with seizures, nor the length of time between seizure onset and surgery (*p* = 0.7731 and *p* = 0.1913, respectively).

Both αβ and γδ T cells in the BIL fractions analyzed (*n* = 14) expressed the early activation marker CD69 indicating that an active immune reaction involving both T cell subtypes was taking place at the time of surgery in the affected cortical hemisphere of these patients. The percent of activated αβ and γδ T cells was highly correlated (Fig. 1d). CD69 expression by infiltrating T cells was confirmed by immunocytochemistry using a second CD69 antibody (Additional file 2: Figure S1).

**Fig. 2:**
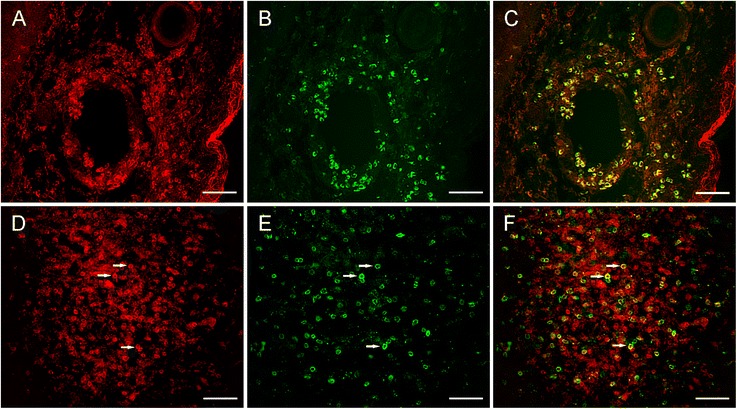
Identification of γδ T cells in RE brain parenchyma by immunofluorescence microscopy. Sections of cortex from a RE hemispherectomy surgery were co-stained with a polyclonal CD3 antibody and a pan TCR γδ mAb. CD3 staining was visualized with an Alexa Fluor® 568 secondary antibody (**a**. **d**), and TCR γδ staining with an Alexa Fluor® 488 secondary antibody (**b**. **e**). **c**, **f** Merged images. *Scale bars* correspond to 100 μm

Figure 2 shows two examples of the distribution of γδ T cells in involved cortical tissue. In Fig. 2a, b, perivascular T cells appear to be predominantly γδ T cells, whereas in Fig. 2d, e, γδ T cells form only part of a large cluster of T cells within brain parenchyma. The other T cells in this cluster are likely to be CD8^+^ αβ T cells.

### A restricted repertoire of γδ T cells in RE brain tissue

**Fig. 3:**
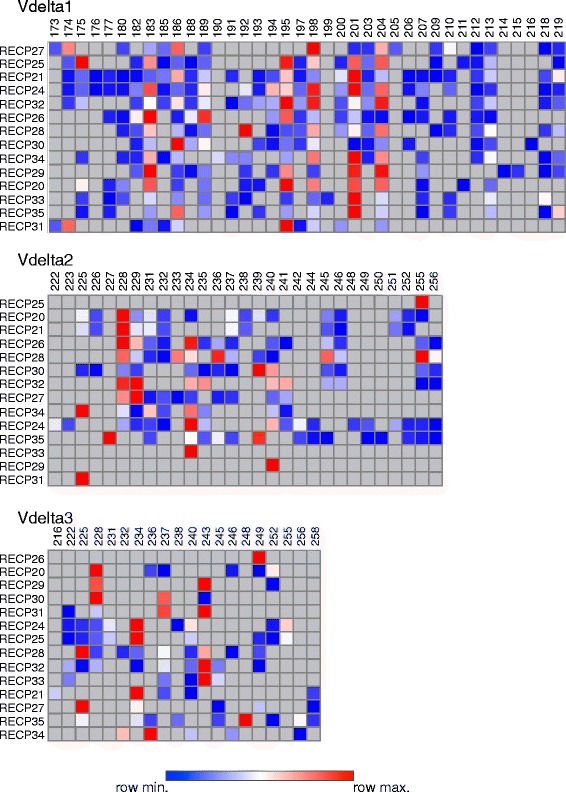
Heat map summarizing the spectratyping of Vδ chains in brain tissue from 14 RE patients. Sequences specific to the three major Vδ chains were amplified by nested PCR and separated by capillary electrophoresis. For each of the 14 RE samples and for each chain-specific primer, the area of each peak corresponding to a different fragment length was expressed as a fraction of the total area under the peaks and converted into a heat map. The data for each Vδ chain were clustered to show which samples were more related to each other based on the fragment lengths, which are listed above each heat map. RNA was extracted from samples of flash frozen brain tissue

**Fig. 4:**
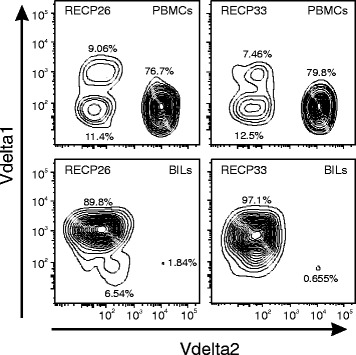
Expression of T cell receptor (TCR) Vδ1 and Vδ2 chains by γδ T cells in brain-infiltrating lymphocytes (BILs) and peripheral blood mononuclear cells (PBMCs) from two RE patients. BILs and PBMCs from two RE cases were stained with CD3, CD4, CD8, TCR αβ, TCR Vδ1-, and TCR Vδ2-specific antibodies. CD3^+^ CD4^−^ CD8^−^ PBMCs are predominantly Vδ2^+^ (76.7 and 79.8 % for RECP26 and RECP33, respectively), whereas most CD3^+^ CD4^−^ CD8^−^ BILs express Vδ1 (89.8 and 97.1 % for RECP26 and RECP33, respectively). The number of CD3+ BILs gated was 1887 for RECP26 and 1516 for RECP33

To gain insight into the T cell receptor (TCR) repertoire of brain-infiltrating γδ T cells, RNA was extracted from flash frozen brain tissue obtained from 14 of the 20 RE cases previously analyzed by flow cytometry. The RNA was transcribed into cDNA and was used as a template to amplify a sequence extending from within the variable regions of the three major TCR δ genes (Vδ1, Vδ2, and Vδ3) to within the common constant region, thus spanning the third complementarity determining region (CDR3), a major determinant of γδ TCR specificity [19]. Fragment lengths differed due to variable D and J segment usage and nucleotide addition and trimming [20]. The heat map in Fig. 3 presents the results of the spectratyping where the different colors denote the relative amounts of each PCR product within a sample. Based on the number of fragments of different sizes, it appears that the repertoire of Vδ chains among the RE samples is relatively restricted. In several cases, only a single amplified product was obtained with the Vδ2- and Vδ3-specific primers (RECP25, RECP29, RECP31, RECP33 for Vδ2, and RECP26 for Vδ3) indicating the presence of single clones. These products were sequenced and, as expected from the size differences, encoded different CDR3 sequences (Additional file 3: Table S2). Compared to the number of Vδ1 fragments, there were fewer Vδ2- or Vδ3-specific products in each sample; several Vδ2 or Vδ3 fragments of the same size were found in over half of the samples. Four of the Vδ1 fragments (183, 186, 198, and 204 bp) were found in all of the samples, and a further three Vδ1 fragments (189, 195, and 201 bp) were found in 13/14 samples. One or more of these seven products were highly expressed in all of the samples, suggesting that they may represent the dominant Vδ1-containing γδ T cell clones in these RE cases. Staining BILs from two of the RE cases (RECP26 and RECP33) with Vδ1- and Vδ2-specific antibodies showed that ≥90 % of the γδ T cells expressed Vδ1, suggesting that δ1 clones may be highly represented in the populations of γδ T cells in RE brain tissue. As expected, the corresponding PBMCs were ≥77 % Vδ2^+^ (Fig. 4) [21].

### Identical Vδ1 CDR3 sequences in RE brain tissue

**Fig. 5:**
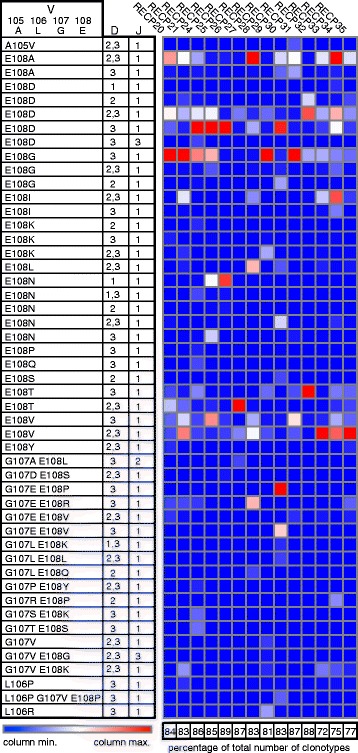
Frequency of δ1 clonotypes present at >1 % in RE brain tissue. PCR fragments amplified with a Vδ1-specific forward primer were sequenced on a MiSeq DNA sequencing platform. Sequences were assigned to individual T cell clonotypes based on three criteria: (1) nucleotides coding for the last four amino acids of the variable (*V*) region, which contribute to the third complementarity determining region (the IMGT algorithm lists amino acid changes with respect to a reference Vδ1 sequence, which is shown in the figure, (2) the number and identity of diversity (*D*) gene segments, and (3) the identity of the joining (*J*) gene sequences. There are three possible D gene segments and four possible J gene segments. Clonotypes were selected from each sample that comprised >1 % of the total repertoire in each sample. Collectively, these 49 clonotypes made up more than 70 % of the repertoire of γδ T cells expressing Vδ1 in each of the RE brain tissue samples. Of these, several dominant clones were evident (*bold-faced type*). A heat map was generated from the quantitative data; the *vertical columns* show the relative abundance of each clonotype in each sample. The data are clustered to show which cases have the most similar repertoires. Additional file 3: Table S3 shows the quantitative data

**Fig. 6:**
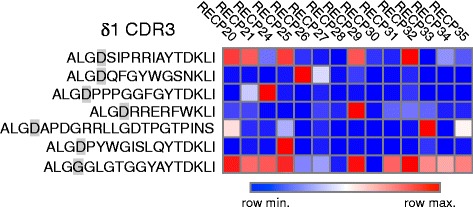
Identical Vδ1 CDR3 sequences in dominant clonotypes present in RE brain tissue. CDR3 sequences of four dominant clonotypes were analyzed, and the most frequent CDR3 sequence in each sample was determined for each clonotype. Identical sequences were found in every sample for three of the clonotypes. The *shaded amino acid* is the last residue encoded in the V gene segment. A heat map was generated from the quantitative data; the *rows* show the relative abundance of each CDR3 sequence in each sample. Additional file 4: Table S4 provides the quantitative data

All of the Vδ1-specific PCR products were sequenced. DNA sequences were curated by submitting them to the IMGT/HighV-QEST web portal [14]. Several thousand different δ1 clonotypes were identified in each sample as defined by differences in the amino acids in the V gene segment that contribute to CDR3 (amino acids 105–108 according to the IMGT standardized numbering system) and by D and J gene segment usage [20]. Strikingly, less than 20 clonotypes were found at a frequency of >1 % in each sample, and these accounted for 64–83 % of the clonotypes in each sample (Table 2). In order to tabulate the frequency of each clonotype in every sample and to determine the extent of overlap, a matrix was constructed for all of the clonotypes found at a frequency of >1 %. As shown in Fig. 5 and in Additional file 4: Table S3, 49 different clonotypes accounted for all of the clonotypes found at >1 % frequency in all of the samples. This indicates that there may be common antigenic targets recognized by these relatively abundant γδ T cell clones. To obtain further evidence for this supposition, the CDR3 amino acid sequences of four dominant clonotypes (Fig. 5 bold-faced type) were examined. Each clonotype comprised several thousand different CDR3 sequences as a result of nucleotide addition and trimming during somatic rearrangement of the TCR gene segments. For each of the four clonotypes, the most frequent CDR3 sequence in each sample was tabulated and compared. For three of the clonotypes, the same CDR3 sequences were found in all of the samples, albeit at varying frequencies (Fig. 6; Additional file 5: Table S4). Strikingly, the most frequent CDR3 sequence in one of the clonotypes was the same in all of the samples (Fig. 6; Additional file 5: Table S4). These CDR3 sequences were not found in a BLAST search of the IMGT database.

**Table 2:** Evidence for a limited number of highly expressed δ1 clonotypes in each RE brain specimen

Case id	No. of clonotypes >1 %	Percent of total no. of clonotypes
RECP20	12	79.1
RECP21	13	78.05
RECP24	16	80.31
RECP25	8	77.8
RECP26	9	83.39
RECP27	6	81.07
RECP28	9	78.87
RECP29	11	76.51
RECP30	14	77.67
RECP31	10	83.35
RECP32	6	83.17
RECP33	9	64.41
RECP34	15	72.05
RECP35	14	72.33

Sequences were assigned to individual clonotypes based on amino acid changes in the residues of the V gene segment that contribute to the CDR3 and D and J gene segment usage. The frequency of each clonotype was calculated. The number of clonotypes >1 % of the total were tabulated, and the frequencies of these clonotypes were summated to give the percentage of the total number of clonotypes

### The identical δ1 CDR3 sequences are detected in dysplastic cortical tissue

**Fig. 7:**
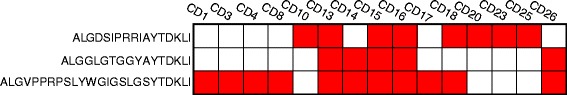
Vδ1 CDR3 sequences in dominant clonotypes are detectable in resected cortical dysplasia brain tissue. Primers were designed based on the most prevalent DNA sequences that specified the CDR3 regions of three of the dominant δ1 clonotypes identified in RE brain tissue. PCR products were sequenced. A *red square* denotes the presence of a sequence, and an *open square* indicates its absence. Aligned DNA sequences are shown in Additional file 8: Figure S2

To determine whether the dominant δ1 clones that we had identified were specific to RE, we designed CDR3 sequence specific PCR primers. We selected the three dominant clonotypes in which a single CDR3 amino sequence predominated (Additional file 5: Table S4). DNA sequences were ranked according to their frequency of occurrence in each RE sample. For each CDR3, a PCR primer was synthesized based on the DNA sequence that was found at the highest frequency in the majority of the samples (Additional file 6: Table S5). We tested the primers on RNA extracted from brain specimens from 15 focal cortical dysplasia (FCD) type I surgical cases [22] (Additional file 7: Table S6). Markers of inflammation, including T cells, have been previously detected in dysplastic brain tissue [18, 23]. With a median age at surgery of 10 years (range 0.8–19 years), the FCD cases were in approximately the same age range as the RE cases (Additional file 7: Table S6). As summarized in Fig. 7 and Additional file 8: Figure S2, sequences identical to those found in the RE samples were detected, demonstrating that the δ1 clones are not unique to RE.

## Discussion

With the participation of eleven other institutions, we accrued fresh brain specimens from 20 RE surgeries over a 2.5-year period. We have carried out an initial characterization of T cells isolated from these specimens in comparison with T cells in peripheral blood from the same patients. Several new and confirmatory observations were made. The profile of brain-infiltrating CD3^+^ T cells was relatively consistent, comprising mostly CD8^+^ αβ and CD4^−^ CD8^−^ γδ T cells with fewer CD4^+^ αβ T cells, whereas the profile of peripheral blood, collected at the time of surgery, was more variable. The predominance of CD8^+^ T cells in the BIL populations agrees with published immunohistochemistry results [12, 16–18], but the presence of γδ T cells is a new finding and has implications for the immune response in RE. This T cell subtype acts like an innate immune cell and plays an important role in both tumor and pathogen immune surveillance [24, 25]. γδ T cells are mainly found in the skin, gut, lungs, and genitourinary tract and normally comprise only a small fraction of circulating T lymphocytes [26, 27]. Unlike αβ T cells, γδ T cells recognize intact antigens and are not dependent on antigen presentation by classical MHC molecules [19, 28]. They recognize transformed and infected cells via NKG2D- and TCR-dependent binding [29]. Stress-induced self-proteins related to MHC molecules, lipid moieties bound to CD1 proteins, heat shock proteins, and phosphorylated metabolites made by certain pathogens and tumor cells are recognized by γδ T cells [19, 28].

Vδ spectratyping showed that γδ T cells in RE brain tissue were oligoclonal and revealed the presence of dominant clonotypes. Vβ spectratyping has also provided evidence for oligoclonality of αβ T cells in RE [30, 31]. Strikingly, the same CDR3 sequences in four abundant δ1 clonotypes were highly represented in every sample. This finding strongly indicates that in all of the RE cases analyzed, and possibly in all RE cases, there are clones of γδ T cells that recognize the same epitope(s). In a study of unrelated newborns who had been infected in utero with cytomegalovirus, T cells with identical TCR Vδ1 CDR3 sequences were found in the blood of every infected individual [32]. It is thought that convergent recombination primarily accounts for the development of public T cell clones [33].

The antigen(s) recognized by γδ T cells in RE are currently unknown. As mentioned above, CD1 proteins are ligands for γδ TCRs. The structures of two different δ1 TCRs complexed with CD1d-bound sulfatide and CD1d-bound α-galactocerebroside, respectively, were recently determined [34, 35]. Two CDR3 motifs, YWG and TDK, that were found to directly interact with residues in CD1d are present in several of the clones that we sequenced (Fig. 6), suggesting the possibility that some γδ T cells in RE brain may bind CD1d. In addition, one of the prevalent CDR3 sequences that we identified contains the heptapeptide motif (AYTDKLI) that is also found in a δ1-containing TCR that binds a stress regulated, MHC class I related molecule, MHC class I polypeptide related sequence A (MICA) [36]. It should be possible to derive clonal lines of γδ T cells from the RE BIL fractions and use binding assays to determine if they interact with these non-classical MHC class I molecules.

Using PCR primers designed to amplify the δ1 CDR3 region from three of the dominant clones, we detected the same sequences in dysplastic brain tissue. The PCR detection method used was not quantitative; thus, we do not yet know how prevalent these δ1 clones are in FCD brain. Further analysis of the γδ T cell repertoire in FCD is clearly warranted and will be the subject of future studies. The finding of γδ TCR sequences is not entirely unexpected as the presence of T cells in FCD brain has been reported [23]. Finding the same δ1 clones in RE and FCD is also consistent with reports of dual pathology [37–42]. Whether the γδ T cell clones that we have identified are only associated with inflammatory events in RE and FCD remains to be determined. If they can be detected in peripheral blood, then they might be useful as a diagnostic indicator of RE, FCD, or brain inflammation in general.

Both RE and FCD are associated with seizures, which have been shown to promote an inflammatory reaction in the brain [43]. γδ T cells are likely to be among the first immune cells to cross the blood brain barrier in response to pro-inflammatory cytokines such as IL-1β and IL-18 released by inflammasomes [43, 44]. The presence of inflammasomes associated with microglia in RE brain tissue has recently been described [45]. In response to inflammatory cytokines, γδ T cells can act as an innate immune cell and release inflammatory cytokines in particular IL-17 without TCR engagement potentially perpetuating an inflammatory reaction [46].

In RE, γδ T cells may provide a link between inflammation in the brain and an adaptive immune response involving antigen-specific CD8^+^ T cells. In multiple sclerosis plaques and psoriatic lesions, γδ T cells are predominantly IL-17^+^, linking this functional subtype to autoimmunity [46–48]. On the other hand, they can act as an adaptive immune cell and bind a cognate antigen and produce IFN-γ [49]. Neurons and astrocytes that express specific stress-induced autoantigens might be directly engaged by Th1-polarized γδ T cells. In mice, the costimulatory receptor CD27 is expressed by IFN-γ^+^ but not IL-17^+^ γδ T cells [50]. Immunostaining BILs with CD27 antibodies may allow us to differentiate between these two functional types of γδ T cells in RE brain tissue. Th1 cytokines such as IFN-γ released by γδ T cells would also be expected to promote a cytotoxic CD8^+^ αβ T cell response [51]. IFN-γ increases MHC class I on neurons, which would render them selectively vulnerable to CD8^+^ cytotoxic αβ T cells [52]. γδ T cells may also play an immunosuppressive role depending upon the stage of the disease, as appears be the case in multiple sclerosis [47]. Multiple roles for γδ T cells in RE might explain why the percentage of γδ T cells in CD3^+^ BILs isolated at the time of surgery appeared to be unrelated to the length of time between seizure onset and surgery.

The cause of RE is unknown. A role for predisposing genetic factors that would influence the outcome of an immune response resulting from seizure-induced inflammation has been proposed [53]. An infectious agent may also be involved in the etiology of RE [4–7, 53, 54], which would fit with the known role of γδ T cells in pathogen surveillance. In a separate RNAseq study, we have looked for evidence of a persistent viral infection in resected RE brain tissue but have not found any gene transcripts encoded by known viruses (unpublished results). In the absence of a persistent infection, RE could involve T cells that recognize an epitope common to both a foreign and a self-antigen, T cells that express dual receptors, or epitope spreading [55–58].

## Conclusions

The cellular immune response in RE involves both classical αβ T cells and non-classical γδ T cells. The γδ T cells appear to be clonally restricted, and prevalent clones may recognize a common antigen(s), possibly a self-antigen associated with stressed cells. γδ T cells may facilitate the activation of auto-reactive αβ T cells by releasing pro-inflammatory cytokines and promoting antigen presentation. The presence of the identical δ1 subtype clones in CD brain implies the involvement of a common inflammatory pathway in both diseases.

## Additional files

Additional file 1: Table S1. Phenotypes of T cells in BIL and PBMC populations from individual RE patients. Additional file 2: Figure S1. CD69 expression by T cells in resected RE brain tissue. Sections of cortex from three RE surgery cases (A, B RECP27; C, D RECP34; D, E RECP37) were stained with either a CD3 polyclonal antibody (A, C, E) or a CD69 mAb (B, D, F). Clusters of T cells, identified by CD3 immunostaining, contain CD69-positive cells. Insets show higher magnification of cells in the upper clusters in each panel (arrows). Scale bars correspond to 200 and 25 micrometers (insets). Additional file 3: Table S2. CDR3 sequences of Vδ2 and Vδ3 clones unique to individual RE cases. Additional file 4: Table S3. Frequency of overlapping clonotypes in each sample as a percentage of the total number of clonotypes in each sample. Additional file 5: Table S4. Frequency of identical CDR3 sequences for dominant clonotypes found in every sample. Additional file 6: Table S5. Frequency of identical CDR3 DNA sequences for dominant clonotypes found in every sample. Additional file 7: Table S6. Clinical data from 15 Focal cortical dysplasia patients. Additional file 8: Figure S2. Alignment of clone-specific CDR3 sequences from FCD cases.

## Abbreviations

BILs: brain-infiltrating lymphocytes
CDR3: third complementarity determining region
FCD: focal cortical dysplasia
IFN-γ: interferon-gamma
MHC: major histocompatibility complex
MICA: MHC class I polypeptide related sequence A
PBMCs: peripheral blood mononuclear cells
RE: Rasmussen encephalitis
TCR: T cell receptor
