# Supplementary material for: Evidence for the involvement of gamma delta T cells in the immune response in Rasmussen encephalitis
Source: J Neuroinflammation. 2015 Jul 19;12:134. doi: 10.1186/s12974-015-0352-2 (PMC4506578; doi:10.1186/s12974-015-0352-2)
Supplement: Additional file 1: Table S1. — Phenotypes of T cells in BIL and PBMC populations from individual RE patients. [file 12974_2015_352_MOESM1_ESM.docx]

Table S1: Phenotypes of T cells in BIL and PBMC populations from individual RE patients (percent CD3^+^ cells)

|  | BILs | | | | | | | | | PBMCs | | | | | | |
| --- | --- | --- | --- | --- | --- | --- | --- | --- | --- | --- | --- | --- | --- | --- | --- | --- |
| CASE | CD4^+^αβ^+^ | CD4^+^γδ^+^ | CD8^+^αβ^+^ | CD8^+^γδ^+^ | CD4^-^CD8^-^αβ^+^ | CD4^-^CD8^-^γδ^+^ | αβ^+^: γδ^+^ | CD69^+^αβ^+^ | CD69^+^γδ^+^ | CD4^+^αβ^+^ | CD4^+^γδ^+^ | CD8^+^αβ^+^ | CD8^+^γδ^+^ | CD4^-^CD8^-^αβ^+^ | CD4^-^CD8^-^γδ^+^ | αβ^+^: γδ^+^ |
| RECP20 | 13.21 | 0.51 | 20.74 | 22.09 | 0.94 | 37.98 | 0.58 | 12.8 | 13.6 | 37.93 | 0.22 | 46.46 | 0.68 | 1.43 | 10.46 | 7.56 |
| RECP21 | 6.48 | 0.93 | 37.93 | 5.57 | 3.70 | 37.00 | 1.11 | n.d. | n.d | 42.03 | 0.79 | 27.96 | 0.65 | 1.00 | 24.92 | 2.69 |
| RECP24 | 9.02 | 0.21 | 40.62 | 2.98 | 21.23 | 20.45 | 3.00 | n.d. | n.d. | 56.00 | 0.00 | 22.38 | 2.60 | 0.00 | 11.54 | 5.55 |
| RECP25 | 9.25 | 0.00 | 63.49 | 4.05 | 0.36 | 15.35 | 3.77 | n.d. | n.d. | 46.49 | 0.91 | 36.25 | 1.98 | 0.00 | 5.39 | 9.98 |
| RECP26 | 7.78 | 0.80 | 53.23 | 10.66 | 0.00 | 22.88 | 1.78 | 27.3 | 31.1 | 59.27 | 0.63 | 25.55 | 0.01 | 0.00 | 8.40 | 9.38 |
| RECP27 | 27.09 | 0.77 | 43.25 | 9.21 | 2.44 | 14.14 | 3.02 | 30.2 | 38.8 | 62.72 | 0.49 | 24.53 | 1.08 | 1.53 | 8.13 | 9.15 |
| RECP28 | 6.15 | 0.00 | 50.81 | 0.85 | 0.00 | 30.49 | 1.82 | 43.5 | 27.6 | 58.50 | 1.32 | 24.75 | 0.00 | 0.17 | 10.96 | 6.79 |
| RECP29 | 19.94 | 0.00 | 24.53 | 2.16 | 0.00 | 30.93 | 1.34 | 90.7 | 98.6 | 64.30 | 0.00 | 19.58 | 1.09 | 1.09 | 10.34 | 7.43 |
| RECP30 | 11.56 | 0.00 | 47.31 | 8.58 | 0.00 | 15.20 | 2.48 | 79.8 | 82.8 | 47.03 | 0.00 | 29.37 | 2.18 | 1.86 | 14.50 | 4.69 |
| RECP31 | 16.34 | 0.00 | 60.14 | 6.86 | 1.61 | 8.13 | 5.21 | 40.4 | 50.6 | 44.82 | 0.00 | 30.20 | 1.27 | 1.90 | 16.27 | 4.39 |
| RECP32 | 19.11 | 0.19 | 51.27 | 4.45 | 0.97 | 22.04 | 2.67 | 51.4 | 50 | 24.75 | 0.00 | 59.90 | 0.00 | 1.73 | 11.07 | 7.80 |
| RECP33 | 12.56 | 0.17 | 24.08 | 0.00 | 1.22 | 58.44 | 0.65 | 50.8 | 63.2 | 60.57 | 0.17 | 29.66 | 0.38 | 0.55 | 7.23 | 11.67 |
| RECP34 | 3.94 | 0.56 | 71.74 | 8.95 | 0.00 | 10.26 | 3.83 | 74.7 | 68.9 | 49.21 | 1.40 | 31.75 | 1.52 | 0.91 | 12.82 | 5.20 |
| RECP35 | 29.80 | 0.12 | 26.11 | 13.55 | 0.00 | 26.61 | 1.39 | 49.1 | 51 | 56.86 | 0.17 | 33.27 | 0.10 | 0.00 | 4.88 | 17.50 |
| RECP36 | 24.46 | 0.00 | 36.13 | 1.54 | 1.12 | 32.69 | 1.80 | 39.1 | 43.8 | 49.79 | 0.00 | 32.40 | 2.07 | 0.33 | 11.66 | 6.01 |
| RECP37 | 9.68 | 0.00 | 45.65 | 3.70 | 15.30 | 23.30 | 2.62 | 16.4 | 21.2 | 63.02 | 0.00 | 28.85 | 0.00 | 0.00 | 3.41 | 26.96 |
| RECP39 | 6.12 | 0.00 | 32.25 | 10.94 | 11.20 | 36.66 | 1.04 | 26.8 | 30.8 | 34.97 | 0.00 | 53.38 | 0.00 | 0.00 | 5.95 | 14.85 |
| RECP42 | 8.17 | 0.00 | 40.31 | 0.00 | 0.00 | 41.90 | 1.96 | n.d. | n.d. | 46.01 | 0.00 | 35.15 | 1.72 | 0.00 | 9.24 | 7.41 |
| RECP43 | 15.33 | 0.00 | 45.55 | 16.72 | 0.00 | 14.36 | 1.25 | n.d. | n.d. | 39.45 | 0.00 | 40.38 | 1.27 | 2.43 | 5.61 | 11.96 |
| RECP46 | 0.31 | 0.00 | 40.28 | 5.56 | 13.12 | 37.31 | 5.22 | n.d. | n.d. | 72.36 | 0.00 | 21.24 | 0.22 | 0.37 | 2.08 | 40.78 |
| **Median** | **10.62** | **0.00** | **41.93** | **5.56** | **0.95** | **24.95** | **1.89** | **41.95** | **46.9** | **49.50** | **0.00** | **29.93** | **0.88** | **0.46** | **9.79** | **7.68** |

n.d. not determined
