# Supplementary material for: Evidence for the involvement of gamma delta T cells in the immune response in Rasmussen encephalitis
Source: J Neuroinflammation. 2015 Jul 19;12:134. doi: 10.1186/s12974-015-0352-2 (PMC4506578; doi:10.1186/s12974-015-0352-2)
Supplement: Additional file 2: Figure S1. — CD69 expression by T cells in resected RE brain tissue. Sections of cortex from three RE surgery cases (A, B RECP27; C, D RECP34; D, E RECP37) were stained with either a CD3 polyclonal antibody (A, C, E) or a CD69 mAb (B, D, F). Clusters of T cells, identified by CD3 immunostaining, contain CD69-positive cells. Insets show higher magnification of cells in the upper clusters in each panel (arrows). Scale bars correspond to 200 and 25 micrometers (insets). [file 12974_2015_352_MOESM2_ESM.pdf]

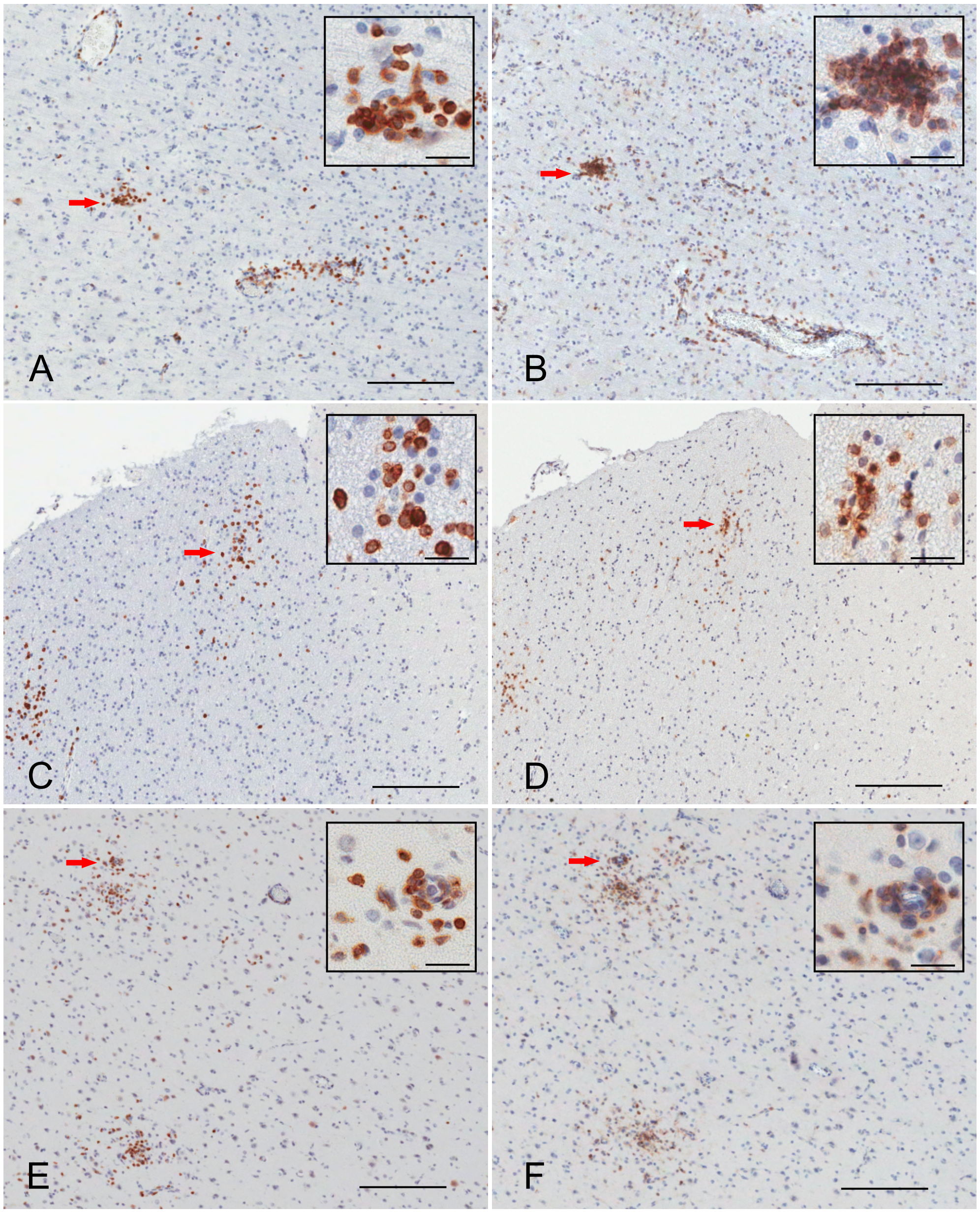

Figure S1: CD69 expression by T cells in resected RE brain tissue. Sections of cortex from three RE surgery cases (A,B RECP27; C,D RECP34; D,E RECP37) were stained with either a CD3 polyclonal antibody (A, C, E) or a CD69 mAb (B, D, F). Clusters of T cells, identified by CD3 immunostaining, contain CD69-positive cells. Insets show higher magnification of cells in the upper clusters in each panel (arrows). Scale bars correspond to 200 μm and 25 μm (insets).
