# Supplementary material for: Evidence for the involvement of gamma delta T cells in the immune response in Rasmussen encephalitis
Source: J Neuroinflammation. 2015 Jul 19;12:134. doi: 10.1186/s12974-015-0352-2 (PMC4506578; doi:10.1186/s12974-015-0352-2)
Supplement: Additional file 3: Table S2. — CDR3 sequences of Vδ2 and Vδ3 clones unique to individual RE cases. [file 12974_2015_352_MOESM3_ESM.docx]

Table S2: CDR3 sequences of Vδ2 and Vδ3 clones unique to individual RE cases

| **Case** | **V** | **D** | **J** | **CDR3** |
| --- | --- | --- | --- | --- |
| RECP25 | 2 | 3 | 1 | ACDTIRKGTFYWGIRGYTDKLI |
| RECP29 | 2 | 3 | 3 | ACDTVLGDSSWDTRQMF |
| RECP31 | 2 | 3 | 1 | ACDTLGDTDKLI |
| RECP33 | 2 | 3 | 1 | ACDSTTGGHDTDKLI |
| RECP26 | 3 | 2 | 1 | AFARPTWVWGPPI |
