# Supplementary material for: Evidence for the involvement of gamma delta T cells in the immune response in Rasmussen encephalitis
Source: J Neuroinflammation. 2015 Jul 19;12:134. doi: 10.1186/s12974-015-0352-2 (PMC4506578; doi:10.1186/s12974-015-0352-2)
Supplement: Additional file 4: Table S3. — Frequency of overlapping clonotypes in each sample as a percentage of the total number of clonotypes in each sample. [file 12974_2015_352_MOESM4_ESM.docx]

Table S3: Frequency of overlapping clonotypes in each sample as a percentage of the total number of clonotypes in each sample.

| **V** | **D** | **J** | **RECP20** | **RECP21** | **RECP24** | **RECP25** | **RECP26** | **RECP27** | **RECP28** | **RECP29** | **RECP30** | **RECP31** | **RECP32** | **RECP33** | **RECP34** | **RECP35** |
| --- | --- | --- | --- | --- | --- | --- | --- | --- | --- | --- | --- | --- | --- | --- | --- | --- |
| A105V | 2,3 | 1 | 0.06 | 0.05 | 0.02 | 0.07 | 0.01 | 0.01 | 0.05 | 0.08 | 0.03 | 4.53 | 0.05 | 0.11 | 0.08 | 0.07 |
| E108A | 2,3 | 1 | 15.24 | 8.74 | 6.18 | 0.83 | 1.90 | 1.18 | 19.99 | 0.80 | 6.88 | 13.74 | 0.59 | 9.67 | 12.42 | 9.83 |
| E108A | 3 | 1 | 0.89 | 0.34 | 0.83 | 0.52 | 0.26 | 0.33 | 0.49 | 0.34 | 3.27 | 8.63 | 0.36 | 0.64 | 0.76 | 0.37 |
| E108D | 1 | 1 | 0.17 | 0.08 | 0.05 | 0.11 | 0.49 | 0.05 | 0.06 | 0.14 | 0.22 | 0.09 | 0.13 | 0.19 | 1.07 | 0.13 |
| E108D | 2 | 1 | 0.68 | 2.04 | 0.71 | 0.51 | 0.33 | 0.29 | 0.11 | 0.42 | 0.23 | 0.12 | 13.04 | 0.43 | 0.35 | 3.10 |
| E108D | 2,3 | 1 | 11.42 | 6.19 | 8.67 | 9.32 | 1.27 | 11.36 | 1.42 | 6.38 | 0.91 | 0.91 | 26.50 | 7.22 | 8.20 | 6.44 |
| E108D | 3 | 1 | 4.06 | 1.03 | 19.21 | 19.43 | 35.96 | 1.52 | 6.80 | 1.16 | 15.47 | 1.08 | 1.08 | 2.41 | 6.15 | 2.55 |
| E108D | 3 | 3 | 0.00 | 0.04 | 2.42 | 0.08 | 0.01 | 0.06 | 0.01 | 0.00 | 0.00 | 0.00 | 0.01 | 0.00 | 0.01 | 0.00 |
| E108G | 3 | 1 | 21.70 | 17.50 | 14.53 | 12.84 | 0.78 | 0.80 | 0.55 | 30.53 | 1.14 | 27.49 | 10.34 | 7.39 | 3.25 | 4.78 |
| E108G | 2,3 | 1 | 0.40 | 0.39 | 0.55 | 3.18 | 0.13 | 0.18 | 0.57 | 0.64 | 1.05 | 0.36 | 0.46 | 0.84 | 2.35 | 0.54 |
| E108G | 2 | 1 | 0.17 | 0.06 | 0.51 | 0.06 | 0.04 | 0.07 | 0.05 | 0.11 | 5.24 | 0.12 | 0.13 | 0.08 | 0.15 | 0.07 |
| E108I | 2,3 | 1 | 0.07 | 7.93 | 0.18 | 0.14 | 0.26 | 0.21 | 5.51 | 0.06 | 0.21 | 0.18 | 0.49 | 8.39 | 10.63 | 2.52 |
| E108I | 3 | 1 | 0.04 | 0.32 | 0.03 | 0.08 | 0.13 | 0.02 | 0.04 | 0.02 | 0.06 | 0.07 | 0.05 | 0.55 | 3.03 | 0.18 |
| E108K | 2 | 1 | 0.06 | 0.05 | 1.51 | 0.07 | 0.06 | 0.05 | 0.02 | 0.05 | 0.02 | 0.01 | 0.03 | 0.03 | 0.03 | 0.02 |
| E108K | 3 | 1 | 1.22 | 0.13 | 0.09 | 0.30 | 0.13 | 0.19 | 0.15 | 0.61 | 0.19 | 0.09 | 0.15 | 0.22 | 0.22 | 0.13 |
| E108K | 2,3 | 1 | 0.21 | 1.56 | 0.14 | 0.13 | 0.00 | 0.28 | 0.23 | 10.06 | 0.16 | 0.10 | 0.12 | 0.39 | 0.00 | 0.42 |
| E108L | 2,3 | 1 | 0.04 | 0.94 | 0.02 | 0.05 | 0.00 | 0.14 | 12.98 | 0.13 | 2.71 | 0.09 | 0.05 | 1.28 | 0.15 | 0.89 |
| E108N | 1 | 1 | 0.04 | 0.08 | 0.31 | 9.65 | 32.21 | 0.71 | 0.26 | 0.24 | 0.08 | 0.25 | 0.13 | 0.15 | 0.03 | 0.04 |
| E108N | 1,3 | 1 | 0.01 | 0.07 | 2.66 | 0.45 | 1.22 | 0.05 | 0.02 | 0.03 | 0.05 | 0.03 | 0.03 | 0.04 | 0.01 | 0.01 |
| E108N | 2 | 1 | 0.01 | 0.01 | 0.02 | 0.36 | 1.35 | 0.03 | 0.02 | 0.02 | 0.01 | 0.03 | 0.03 | 0.03 | 0.01 | 0.01 |
| E108N | 2,3 | 1 | 0.67 | 0.07 | 0.02 | 0.20 | 0.29 | 0.06 | 0.11 | 0.13 | 6.85 | 0.22 | 0.12 | 0.15 | 1.06 | 0.44 |
| E108N | 3 | 1 | 0.11 | 0.19 | 0.34 | 7.79 | 0.22 | 0.17 | 0.08 | 0.09 | 0.25 | 1.26 | 0.12 | 0.18 | 0.12 | 0.24 |
| E108P | 3 | 1 | 0.01 | 0.03 | 1.09 | 0.03 | 0.01 | 0.01 | 0.05 | 0.01 | 0.21 | 0.03 | 0.01 | 0.01 | 0.02 | 0.01 |
| E108Q | 3 | 1 | 0.09 | 0.09 | 2.68 | 0.20 | 0.06 | 0.13 | 0.10 | 0.10 | 0.09 | 0.04 | 0.07 | 0.08 | 0.09 | 0.06 |
| E108S | 2 | 1 | 0.02 | 0.00 | 0.01 | 0.01 | 0.05 | 0.02 | 0.01 | 0.01 | 2.29 | 0.03 | 0.02 | 0.03 | 0.01 | 0.01 |
| E108T | 3 | 2 | 3.03 | 0.71 | 5.04 | 0.21 | 0.71 | 0.24 | 0.04 | 6.40 | 0.13 | 4.18 | 31.09 | 0.42 | 2.95 | 4.40 |
| E108T | 2,3 | 1 | 8.22 | 3.51 | 0.87 | 1.39 | 0.59 | 48.14 | 0.42 | 0.02 | 0.20 | 0.11 | 0.08 | 0.22 | 1.12 | 2.93 |
| E108V | 3 | 1 | 4.23 | 7.34 | 2.53 | 14.20 | 3.66 | 0.70 | 6.86 | 1.08 | 0.60 | 15.43 | 1.13 | 1.56 | 4.19 | 4.38 |
| E108V | 2,3 | 1 | 3.19 | 13.25 | 2.38 | 1.39 | 4.34 | 12.42 | 9.73 | 4.60 | 0.42 | 4.54 | 0.50 | 21.50 | 10.21 | 22.52 |
| E108Y | 2,3 | 1 | 1.79 | 0.00 | 0.07 | 0.11 | 0.04 | 0.07 | 0.05 | 0.07 | 0.06 | 0.04 | 0.06 | 0.19 | 0.11 | 1.68 |
| G107A E108L | 3 | 1 | 0.01 | 0.01 | 0.02 | 0.05 | 0.03 | 6.46 | 0.06 | 0.02 | 0.01 | 0.01 | 0.01 | 0.00 | 0.01 | 0.01 |
| G107D E108S | 2,3 | 1 | 0.00 | 0.00 | 0.00 | 0.06 | 1.49 | 0.04 | 0.01 | 0.01 | 0.01 | 0.00 | 0.01 | 0.00 | 0.00 | 0.00 |
| G107E E108P | 3 | 1 | 0.04 | 0.01 | 0.19 | 0.01 | 0.00 | 0.01 | 0.01 | 0.03 | 16.36 | 0.19 | 0.10 | 0.09 | 0.02 | 0.03 |
| G107E E108R | 3 | 1 | 2.87 | 2.42 | 2.07 | 0.16 | 0.06 | 0.23 | 13.02 | 0.09 | 0.09 | 0.05 | 0.05 | 0.15 | 0.02 | 4.22 |
| G107E  E108V | 2,3 | 1 | 2.14 | 0.46 | 0.00 | 0.07 | 0.02 | 0.06 | 0.02 | 0.02 | 0.23 | 2.44 | 0.04 | 0.86 | 0.03 | 1.37 |
| G107E E108V | 3 | 1 | 0.08 | 0.02 | 0.00 | 0.03 | 0.01 | 0.01 | 0.01 | 0.01 | 9.87 | 0.17 | 0.07 | 0.08 | 0.02 | 0.05 |
| G107L E108K | 1,3 | 1 | 0.00 | 0.00 | 0.00 | 0.00 | 0.00 | 0.00 | 0.00 | 0.01 | 1.47 | 0.02 | 0.02 | 0.01 | 0.00 | 0.01 |
| G107L E108L | 2,3 | 1 | 0.01 | 0.00 | 0.00 | 0.00 | 0.00 | 0.00 | 0.01 | 0.00 | 2.04 | 0.03 | 0.01 | 0.01 | 0.01 | 0.00 |
| G107L E108Q | 2 | 1 | 0.00 | 0.00 | 0.00 | 0.01 | 0.00 | 0.03 | 2.57 | 0.01 | 0.00 | 0.00 | 0.00 | 0.00 | 0.00 | 0.00 |
| G107P E108Y | 2,3 | 1 | 0.00 | 0.04 | 1.38 | 0.05 | 0.00 | 0.01 | 0.01 | 0.00 | 0.00 | 0.00 | 0.01 | 0.00 | 0.00 | 0.00 |
| G107R E108P | 2 | 1 | 0.00 | 0.00 | 0.00 | 0.00 | 0.06 | 0.00 | 0.00 | 0.00 | 0.04 | 0.02 | 0.03 | 0.06 | 3.45 | 0.05 |
| G107S E108K | 3 | 1 | 0.00 | 0.00 | 3.78 | 0.00 | 0.00 | 0.00 | 0.00 | 0.00 | 0.00 | 0.00 | 0.00 | 0.00 | 0.00 | 0.00 |
| G107T E108S | 3 | 1 | 0.00 | 0.11 | 4.18 | 0.15 | 0.03 | 0.01 | 0.01 | 0.00 | 0.00 | 0.01 | 0.02 | 0.00 | 0.00 | 0.00 |
| G107V | 2,3 | 1 | 0.12 | 0.12 | 0.05 | 0.10 | 0.03 | 0.06 | 0.15 | 3.37 | 0.06 | 0.05 | 0.08 | 0.21 | 0.17 | 0.10 |
| G107V E108G | 2,3 | 3 | 0.01 | 0.00 | 0.00 | 0.01 | 0.00 | 0.00 | 0.00 | 1.05 | 0.01 | 0.00 | 0.00 | 0.01 | 0.00 | 0.00 |
| G107V E108K | 2,3 | 1 | 0.02 | 5.02 | 0.06 | 0.05 | 0.23 | 0.05 | 0.02 | 2.45 | 0.05 | 0.07 | 0.06 | 4.99 | 1.97 | 1.60 |
| L106P | 3 | 1 | 0.40 | 1.51 | 0.18 | 0.35 | 0.13 | 0.26 | 0.25 | 0.60 | 0.32 | 0.17 | 0.26 | 0.45 | 0.36 | 0.50 |
| L106P G107V E108P | 3 | 1 | 0.01 | 0.00 | 0.00 | 0.00 | 0.00 | 0.00 | 0.01 | 0.01 | 3.03 | 0.04 | 0.02 | 0.01 | 0.00 | 0.01 |
| L106R | 3 | 1 | 0.34 | 0.21 | 0.11 | 0.44 | 0.13 | 0.27 | 0.18 | 9.42 | 0.33 | 0.15 | 0.35 | 0.30 | 0.20 | 0.19 |
