# Supplementary material for: Evidence for the involvement of gamma delta T cells in the immune response in Rasmussen encephalitis
Source: J Neuroinflammation. 2015 Jul 19;12:134. doi: 10.1186/s12974-015-0352-2 (PMC4506578; doi:10.1186/s12974-015-0352-2)
Supplement: Additional file 5: Table S4. — Frequency of identical CDR3 sequences for dominant clonotypes found in every sample. [file 12974_2015_352_MOESM5_ESM.docx]

Table S4: Frequency of identical CDR3 sequences for dominant clonotypes found in every sample (percentage of the total number of CDR3 sequences for the clonotype in each sample).

| Clonotype | V(E108D) D2,3 J1 | V(E108D) D3 J1 | | | | | V(E108G) D3 J1 | V(E108V) D2,3 J1 |
| --- | --- | --- | --- | --- | --- | --- | --- | --- |
| CDR3 | ALGDSIPRRI  ATDKLI | ALGDQFGYWS  NKLI | ALGDPPPGGF  GTDKLI | ALGDRRERFW  KLI | ALGDAPDGRR  LGDTPGTPINS | ALGDPYWGIS  LQTDKLI | ALGGLGTGGY  AYDKLI | ALGVPPRPSLYW  GIGSLGSYTDKLI |
| RECP20 | 75.76 | 2.41 | 0.18 | 3.66 | 16.59 | 0.15 | 80.72 | 0.00 |
| RECP21 | 71.13 | 2.30 | 30.26 | 3.63 | 2.56 | 2.67 | 71.04 | 73.07 |
| RECP24 | 21.83 | 0.31 | 76.26 | 0.45 | 1.75 | 1.30 | 72.98 | 4.69 |
| RECP25 | 75.94 | 3.41 | 3.49 | 0.27 | 10.10 | 63.38 | 79.21 | 18.48 |
| RECP26 | 11.68 | 79.81 | 0.16 | 0.04 | 0.04 | 0.12 | 38.02 | 2.27 |
| RECP27 | 7.08 | 34.53 | 5.10 | 3.39 | 2.31 | 3.67 | 41.19 | 8.71 |
| RECP28 | 3.82 | 2.31 | 0.74 | 1.99 | 0.04 | 0.28 | 25.47 | 78.26 |
| RECP29 | 72.00 | 9.66 | 2.20 | 26.58 | 0.33 | 1.14 | 82.18 | 42.82 |
| RECP30 | 11.94 | 0.19 | 0.10 | 0.64 | 0.04 | 0.01 | 22.01 | 28.76 |
| RECP31 | 9.26 | 1.63 | 1.53 | 5.26 | 0.13 | 0.07 | 71.81 | 36.61 |
| RECP32 | 84.92 | 6.57 | 8.13 | 6.09 | 0.86 | 0.52 | 83.17 | 31.33 |
| RECP33 | 3.48 | 0.52 | 0.59 | 5.33 | 28.99 | 6.57 | 67.34 | 72.60 |
| RECP34 | 26.97 | 0.27 | 0.06 | 1.63 | 0.06 | 0.02 | 63.28 | 36.87 |
| RECP35 | 17.62 | 0.28 | 0.21 | 2.00 | 15.35 | 2.00 | 68.16 | 64.15 |
