# Supplementary material for: Evidence for the involvement of gamma delta T cells in the immune response in Rasmussen encephalitis
Source: J Neuroinflammation. 2015 Jul 19;12:134. doi: 10.1186/s12974-015-0352-2 (PMC4506578; doi:10.1186/s12974-015-0352-2)
Supplement: Additional file 6: Table S5. — Frequency of identical CDR3 DNA sequences for dominant clonotypes found in every sample. [file 12974_2015_352_MOESM6_ESM.docx]

Table S5: Frequency of identical CDR3 DNA sequences for dominant clonotypes found in every sample (percentage of the total number of DNA sequences specifying the amino acid sequence for the dominant clonotype in each sample).

| Clonotype | V(E108D) D2,3 J 1 | V(E108G) D3 J1 | V(E108V) D2,3 J1 |
| --- | --- | --- | --- |
| CDR3 | ALGDSIPRRIAYTDKLI | ALGGLGTGGYAYTDKLI | ALGVPPRPSLYWGIGSLGSYTDKLI |
|  | 5’ gctcttggggattccattcctaggag  gatagcgtacaccgataaactcatc 3’ | 5’gctcttggggggctaggtactgggg  gatacgcctacaccgataaactcatc 3’ | 5’ gctcttggggtcccgcctcgaccttccctctactgggg  gataggaagcttgggctcgtacaccgataaactcatc 3’ |
| RECP20 | 72.72 | 74.37 | 0.50 |
| RECP21 | 68.34 | 66.91 | 57.85 |
| RECP24 | 21.16 | 61.49 | 3.96 |
| RECP25 | 73.53 | 73.09 | 14.16 |
| RECP26 | 10.97 | 33.77 | 1.72 |
| RECP27 | 17.31 | 35.23 | 6.95 |
| RECP28 | 3.56 | 23.44 | 66.52 |
| RECP29 | 68.87 | 75.85 | 35.65 |
| RECP30 | 10.61 | 19.43 | 22.25 |
| RECP31 | 8.32 | 67.36 | 30.42 |
| RECP32 | 81.66 | 78.19 | 26.41 |
| RECP33 | 3.22 | 63.06 | 60.99 |
| RECP34 | 25.74 | 58.78 | 28.54 |
| RECP35 | 12.25 | 62.73 | 64.15 |
