# Supplementary material for: Evidence for the involvement of gamma delta T cells in the immune response in Rasmussen encephalitis
Source: J Neuroinflammation. 2015 Jul 19;12:134. doi: 10.1186/s12974-015-0352-2 (PMC4506578; doi:10.1186/s12974-015-0352-2)
Supplement: Additional file 7: Table S6. — Clinical data from 15 Focal cortical dysplasia patients. [file 12974_2015_352_MOESM7_ESM.docx]

**Table S6:** Clinical data from 15 cortical dysplasia patients

| **Case ID** | **ILAE type** | **Gender** | **Age at seizure onset (yr)** | **Age at surgery (yr)** | **Hemisphere** |
| --- | --- | --- | --- | --- | --- |
| CD1 | 1a | F | 1.4 | 10 | L |
| CD3 | 1a | F | 1.5 | 10 | L |
| CD4 | 1a | F | 3 | 6.5 | R |
| CD8 | 1a | M | 2 | 14 | L |
| CD10 | 1a | F | 0.1 | 8 | R |
| CD13 | 1c | F | 4.5 | 6 | R |
| CD14 | 1c | M | 0.5 | 2.5 | R |
| CD15 | 1c | M | 0.3 | 0.83 | R |
| CD16 | 1c | M | 6 | 17 | L |
| CD17 | 1c | M | 7 | 19 | L |
| CD18 | 1c | M | 5.5 | 12 | L |
| CD20 | 1c | M | 1.4 | 10 | R |
| CD23 | 1c | M | 0.75 | 1.3 | R |
| CD25 | 1c | M | 0.3 | 1.1 | L |
| CD26 | 1a | M | 0.5 | 10 | R |

ILAE, International League Against Epilepsy
