# Supplementary material for: Evidence for the involvement of gamma delta T cells in the immune response in Rasmussen encephalitis
Source: J Neuroinflammation. 2015 Jul 19;12:134. doi: 10.1186/s12974-015-0352-2 (PMC4506578; doi:10.1186/s12974-015-0352-2)
Supplement: Additional file 8: Figure S2. — Alignment of clone-specific CDR3 sequences from FCD cases. [file 12974_2015_352_MOESM8_ESM.docx]

Figure S2: Alignment of clone-specific CDR3 sequences from CD cases.

**CDR3: ALGDSIPRRIAYTDKLI**

5’ gctcttggggattccattcctaggaggatagcgtacaccgataaactcatc 3’

D 2,3  **|**J 1 →

CD10 GCTCTTGGGG ATTCCATTCC TAGGAGGATA GCGTACACCG ATAAACTCAT CTTTGGAAAA GGAACCCGTG

CD13 GCTCTTGGGG ATTCCATTCC TAGGAGGATA GCGTACACCG ATAAACTCAT CTTTGGAAAA GGAACCCGTG

CD15 GCTCTTGGGG ATTCCATTCC TAGGAGGATA GCGTACACCG ATAAACTCAT CTTTGGAAAA GGAACCCGTG

CD16 GCTCTTGGGG ATTCCATTCC TAGGAGGATA GCGTACACCG ATAAACTCAT CTTTGGAAAA GGAACCCGTG

CD18 GCTCTTGGGG ATTCCATTCC TAGGAGGATA GCGTACACCG ATAAACTCAT CTTTGGAAAA GGAACCCGTG

CD20 GCTCTTGGGG ATTCCATTCC TAGGAGGATA GCGTACACCG ATAAACTCAT CTTTGGAAAA GGAACCCGTG

CD23 ---CTTGGGG ATTCCATTCC TAGGAGGATA GCGTACACCG ATAAACTCAT CTTTGGAAAA GGAACCCGTG

CD25 ----TTGGGG ATTCCATTCC TAGGAGGATA GCGTACACCG ATAAACTCAT CTTTGGAAAA GGAACCCGTG

**|**Constant region →

CD10 TGACTGTGGA ACCAAGAAGT CAGCCTCATA CCAAACCATC CGTTTTTGTC ATGAAAAATG GAACAAATGT

CD13 TGACTGTGGA ACCAAGAAGT CAGCCTCATA CCAAACCATC CGTTTTTGTC ATGAAAAATG GAACAAATGT

CD15 TGACTGTGGA ACCAAGAAGT CAGCCTCATA CCAAACCATC CGTTTTTGTC ATGAAAAATG GAACAAATGT

CD16 TGACTGTGGA ACCAAGAAGT CAGCCTCATA CCAAACCATC CGTTTTTGTC ATGAAAAATG GAACAAATGT

CD18 TGACTGTGGA ACCAAGAAGT CAGCCTCATA CCAAACCATC CGTTTTTGTC ATGAAAAATG GAACAAATGT

CD20 TGACTGTGGA ACCAAGAAGT CAGCCTCATA CCAAACCATC CGTTTTTGTC ATGAAAAATG GAACAAATGT

CD23 TGACTGTGGG ACCAAGAAGT CAGCCTCATA CCAAACCATC CGTTTTTGTC ATGAAAAATG GAACAAATGT

CD25 TGACTGTGGA ACCAAGAAGT CAGCCTCATA CCAAACCATC CGTTTTTGTC ATGAAAAATG GAACAAATGT

**CDR3: ALGGLGTGGYAYTDKLI**

5’ gctcttggggggctaggtactgggggatacgcctacaccgataaactcatc 3’

D 3  **|**J 1 →

CD13 ---------- ---------G GGGATACGCC TACACCGATA AACTCATCTT TGGAAAAGGA ACCCGTGTGA

CD14 CTTGGGGGGC TAGGTACTGG GGGATACGCC TACACCGATA AACTCATCTT TGGAAAAGGA ACCCGTGTGA

CD15 ---------- ----TACTGG GGGATACCCC TACACCGATA AACTCATCTT TGGAAAAGGA ACCCGTGTGA

CD16 ----GGGGGC TAGGTACTGG GGGATACGCC TACACCGATA AACTCATCTT TGGAAAAGGA ACCCGTGTGA

CD27 ---------- --------GG GGGATACGCC TACCCCGATA AACTCATCTT TGGAAAAGGA ACCCGTGTGA

**|**Constant region →

CD13 CTGTGGAACC AAGAAGTCAG CCTCATACCA AACCATCCGT TTTTGTCATG AAAAATGGAA CAAATGTCGC

CD14 CTGTGGAACC AAGAAGTCAG CCTCATACCA AACCATCCGT TTTTGTCATG AAAAATGGAA CAAATGTCGC

CD15 CTGTGGAACC AAGAAGTCAG CCTCATACCA AACCATCCGT TTTTGTCATG AAAAATGGAA CAAATGTCGC

CD16 CTGTGGAACC AAGAAGTCAG CCTCATACCA AACCATCCGT TTTTGTCATG AAAAATGGAA CAAATGTCGC

CD26 CTGTGGAACC AAGAAGTCAG CCTCATACCA AACCATCCGT TTTTGTCATG AAAAATGGAA CAAATGTCGC

**CDR3: ALGVPPRPSLYWGIGSLGSYTDKLI**

5’ gctcttggggtcccgcctcgaccttccctctactgggggataggaagcttgggctcgtacaccgataaactcatc 3’

D 2,3  **|**J 1 →

CD1 GACCTTCCCT CTACTGGGGG ATAGGAAGCT TGGGCTCGTA CACCGATAAA CTCATCTTTG GAAAAGGAAC

CD3 GACCTTCCCT CTACTGGGGG ATAGGAAGCT TGGGCTCGTA CACCGATAAA CTCATCTTTG GAAAAGGAAC

CD4 GACCTTCCCT CTACTGGGGG ATAGGAAGCT TGGGCTCGTA CACCGATAAA CTCATCTTTG GAAAAGGAAC

CD8 ---CTTCCCT CTACTGGGGG ATAGGAAGCT TGGGCTCGTA CACCGATAGA CTCATCTTTG GAAAAGGAAC

CD13 -ACCTTCCCT CTACTGGGGG ATAGGAAGCT TGGGCTCGTA CACCGATAAA CTCATCTTTG GAAAAGGAAC

CD14 ---CTTCCCT CTACTGGGGG ATAGGAAGCT TGGGCTCGTA CACCGATAAA CTCATCTTTG GAAAAGGAAC

CD15 GACCTTCCCT CTACTGGGGG ATAGGAAGCT TGGGCTCGTA CACCGATAAA CTCATCTTTG GAAAAGGAAC

CD16 GACCTTCCCT CTACTGGGGG ATAGGAAGCT TGGGCTCGTA CACCGATAAA CTCATCTTTG GAAAAGGAAC

CD17 GACCTTCCCT CTACTGGGGG ATAGGAAGCT TGGGCTCGTA CACCGATAAA CTCATCTTTG GAAAAGGAAC

CD18 GACCTTCCCT CTACTGGGGG ATAGGAAGCT TGGGCTCGTA CACCGATAAA CTCATCTTTG GAAAAGGAAC

CD26 GACCTTCCCT CTACTGGGGG ATAGGAAGCT TGGGCTCGTA CACCGATAAA CTCATCTTTG GAAAAGGAAC

**|**Constant region →

CD1 CCGTGTGACT GTGGAACCAA GAAGTCAGCC TCATACCAAA CCATCCGTTT TTGTCATGAA GAATGGAACA

CD3 CCGTGTGACT GTGGAACCAA GAAGTCAGCC TCATACCAAA CCATCCGTTT TTGTCATGAA GAATGGAACA

CD4 CCGTGTGACT GTGGAACCAA GAAGTCAGCC TCATACCAAA CCATCCGTTT TTGTCGTGAA AAATGGAACA

CD8 CCGTGTGACT GTGGAACCAA GAAGTCAGCC TCATACCAAA CCATCCGTTT TTGTCATGAA AAATGGAACA

CD13 CCGTGTGACT GTGGAACCAA GAAGTCAGCC TCATACCAAA CCATCCGTTT TTGTCATGAA AAATGGAACA

CD14 CCGTGTGACT GTGGAACCAA GAAGTCAGCC TCATACCAAA CCATCCGTTT TTGTCATGAA AAATGGAACA

CD15 CCGTGTGACT GTGGAACCAA GAAGTCAGCC TCATACCAAA CCATCCGTTT TTGTCATGAA AAATGGAACA

CD16 CCGTGTGACT GTGGAACCAA GAAGTCAGCC TCATACCAAA CCATCCGTTT TTGTCATGAA AAATGGAACA

CD17 CCGTGTGACT GTGGAACCAA GAAGTCAGCC TCATACCAAA CCATCCGTTT TTGTCATGAA AAATGGAACA

CD18 CCGTGTGACT GTGGAACCAA GAAGTCAGCC TCATACCAAA CCATCCGTTT TTGTCATGAA AAATGGAACA

CD26 CCGTGTGACT GTGGAACCAA GAAGTCAGCC TCATACCAAA CCATCCGTTT TTGTCATGAA AAATGGAACA
